# Adaptive multimodal fusion via Gated Parallel Mamba architecture for ultra-high-precision stroke lesion segmentation in medical imaging

**DOI:** 10.1371/journal.pdig.0001517

**Published:** 2026-07-17

**Authors:** Runnan He, Leshui Dong, Shuang Xia, Chen Cao, Youwei Wang, Meijun Pang, Kuo Zhang, Xiuyun Liu, Dong Ming, Mark Haacke

**Affiliations:** 1 Medical School of Tianjin University, Nankai District, Tianjin, China; 2 Department of Radiology Tianjin First Central Hospital, Nankai District, Tianjin, China; 3 Tianjin Huanhu Hospital, Jinnan District, Tianjin, China; 4 State Key Laboratory of Advanced Medical Materials and Devices, Tianjin, China; 5 Haihe Laboratory of Brain-Computer Interaction and Human-Machine Integration, Tianjin, China; 6 School of Pharmaceutical Science and Technology Tianjin University, Nankai District, Tianjin, China; 7 Department of Radiology, Ruijin Hospital, Shanghai Jiaotong University, Shanghai, China; University of Illinois Urbana-Champaign, UNITED STATES OF AMERICA

## Abstract

The accurate delineation of ischemic stroke lesions in magnetic resonance imaging (MRI) is impeded by heterogeneous lesion morphology and the computational expense of modeling global context in three‑dimensional data. In cerebral infarction assessment, diffusion‑weighted imaging, apparent diffusion coefficient, T2‑weighted imaging and T2star sequences (including susceptibility weighted image processing) each offer complementary information, yet existing fusion strategies often fail to adapt to missing modalities or capture long‑range dependencies efficiently. Here we present GPMNet, a lightweight convolutional framework that integrates an adaptive multimodal feature fusion module—employing dynamic cross‑attention to spatially weight and merge signals from all four MRI sequences—and a gated parallel state‑space module that models global voxel interactions in linear time via dual gated branches. We trained the network end-to-end on the ATLAS R2.0 dataset and our own dataset collected at HuanHu Hospital (Tianjin, China), labeled as HHD. The training used a combined Dice–binary cross-entropy and TOPK10 loss, and the outputs were refined using ensemble inference and connected-domain filtering. GPMNet achieved Dice coefficients of 0.6604 and 0.7171 on the two cohorts respectively, achieving superior results compared to other state-of-the-art algorithms. Moreover, the Grad-CAM–based interpretability analysis confirms that the model’s attention corresponds to true ischemic areas across modalities, offering visual evidence of its diagnostic reliability and enhancing the transparency of the segmentation process. Our approach delivered rapid, high‑precision stroke segmentation and establishes a scalable paradigm for resource‑efficient clinical imaging applications.

## Author summary

Stroke is a leading cause of death and long-term disability worldwide. Doctors rely on brain scans, such as MRI, to identify damaged areas, but analyzing these images can be time-consuming and challenging. Different types of MRI scans show different aspects of the brain, and combining this information accurately is not straightforward. In this study, we developed a new computer-based method to automatically detect stroke-related brain damage from multiple types of MRI images. Our approach learns how to combine information from different scans and can identify both small and large lesions more accurately than existing methods. We tested our method on both public data and real clinical cases, where it showed improved performance. Importantly, the system can also highlight the regions it focuses on, helping doctors understand and trust its results. This work may support faster and more reliable stroke assessment, ultimately aiding clinical decision-making and improving patient care.

## 1. Introduction

Stroke is one of the most prevalent cerebrovascular diseases globally and is a leading cause of both morbidity and mortality, particularly requiring timely critical care to optimize patient outcomes. In the United States alone, approximately 795,000 individuals experience either a new or recurrent stroke annually, with stroke prevalence in adults estimated at around 3% [1]. Magnetic Resonance Imaging (MRI) plays a vital role in stroke detection, and accurate lesion segmentation enables precise lesion localization, which is extremely important in clinical practice [2,3]. Currently, the predominant segmentation method is mainly through manual labeling by experienced clinicians, which is time-consuming and labor-intensive, and greatly relies on the clinicians’ expertise [4]. Developing an automatic algorithm for precise stroke lesion segmentation is an urgent need, facing significant challenges due to the high variability of strokes. These challenges include not only diverse lesion locations, shapes, and sizes, but also the often-subtle intensity contrast between the lesion and surrounding healthy tissue, and the difficulty of effectively fusing complementary information from multiple MRI modalities.

In recent years, with progress in machine learning and deep learning techniques, convolutional neural networks (CNNs) have gained prominence in medical image analysis [5,6]. U-Net, a CNN-based method, employs an encoder-decoder architecture to extract both global and local features from images, making it quite popular in brain disease diagnosis [7]. However, CNNs have difficulties in capturing long-range dependencies because its convolutional layers are only able to extract local pixel features [8]. Conversely, Transformer-based models overcome the above problems due to their self-attention mechanism [9]. Various Transformer-based methods, such as MLiRA-Net and Hybrid CNN-Transformer Network, have been widely adopted in stroke segmentation [10,11]. Despite their excellent performance in global modeling and long-range dependency feature extraction, the self-attention mechanism of Transformers scales quadratically with the size of the input image, rendering them computationally demanding, particularly for 3D high-resolution medical images.

Recently, structured state-space sequence (S4) models [12] have demonstrated promising advantages in modeling lengthy sequential data and achieved state-of-the-art performance in computer vision tasks [13]. Mamba [14], characterized by its selective mechanism and hardware-aware features, maintains linear computational complexity while effectively capturing long-range dependencies. These features and advantages serve as the impetus for exploring the potential of Mamba in stroke segmentation. However, the application of Mamba in stroke lesion segmentation has not been explored. In this study, we make the first attempt to introduce Mamba structures into medical image segmentation tasks, especially for the automatic detection of stroke lesions. We aim to address two central problems in current approaches. On the one hand, convolutional networks have difficulty in effectively modeling long-range spatial dependencies in images. On the other hand, the computational overhead of the Transformer approach is too high when processing 3D medical images. We have embedded Mamba into a gated parallel structure, which not only preserves its long-range modeling capability, but also regulates and compresses the feature information through the gating mechanism, thus reducing the computational burden and enhancing the model’s ability to represent complex lesion features in a more clinically relevant way.

Despite the success of existing multimodal medical image segmentation models, most approaches either treat the different modalities equally (e.g., through early connectivity) or treat them separately, lacking an effective mechanism to capture the semantic dependencies between the different modalities. Such fusion strategies are insufficient to fully utilize the potential of multimodal MRI data, especially when the importance of each modality varies spatially and contextually. To address this limitation, we propose to use the Adaptive Multimodal Feature Fusion (AMF) module, which dynamically integrates in-depth features from multiple MRI modalities through a cross-attention mechanism. This design allows each modality to act as both a querier and a responder, resulting in context-aware asymmetric fusion that enhances complementary information and suppresses redundancy. In addition, the AMF is robust to modality deletion and can adapt to single or multi-modal inputs without structural adjustments.

In the current study, we developed an automatic segmentation model that integrates Mamba to achieve precise voxel segmentation of stroke lesions. The main contributions to this analysis include: (1) Adopting an Adaptive Multimodal Feature Fusion (AMF) module, which enables the fusion of multimodal features through the valence attention mechanism. (2) Developing a stroke segmentation method based on Gated Parallel Mamba Network (GPMNet), which is able to efficiently capture long-range dependencies in images and incorporates the feature extraction capabilities of CNN convolutional layers. To our knowledge, this is the first application of Mamba structures in stroke segmentation. (3) Training the proposed model based on DiceBCE loss and TOPK10 loss. Subsequently, we combined the results of both training approaches to achieve accurate segmentation results and enhance the robustness of the model. And, finally, (4) evaluating the proposed method using various datasets, and compare our model with other state-of-the-art algorithms. Furthermore, to enhance the transparency of the decision-making process, we introduce Grad-CAM–based visual interpretability analysis, enabling the examination of the spatial attention distribution of GPMNet and providing visual evidence of model reliability.

## 2. Related work

### 2.1 Stroke lesion segmentation

In recent years, CNNs [15,16] have demonstrated remarkable efficacy in medical image segmentation tasks. Several models based on the U-Net [7] architecture have exhibited promise in stroke lesion segmentation. For example, Zhou et al. introduced D-UNet [17], integrating 2D and 3D convolutionally extracted features for superior performance over 2D CNNs. Qi et al. proposed X-Net [18], employing depth-separable convolutions for denser contextual information extraction and enhanced segmentation accuracy. Additionally, Kumar et al. devised CSNet [19], a cascade architecture model combining parameter sharing of U-Net with the repeated generation of self-similar objects strategy of Fractal-Net [20] to enhance the segmentation accuracy. However, the limitations of convolutional operations hinder the network’s ability to capture long-range dependencies in images. Recently, several studies based on the Transformer architecture have addressed this limitation effectively [21]. For instance, Wu et al. introduced W-Net [22], a hybrid network architecture comprising CNN and Transformer components. This model incorporates boundary deformation and constraint modules to address challenging segmentation of stroke lesion boundaries, particularly in the presence of background noise. METrans [23] utilized a multi-scale encoder for feature extraction, feeding the feature map into a Transformer to capture global features, yielding state-of-the-art results across multiple datasets. Additionally, Luo et al. [24] employed a Transformer to model global contextual information, enabling high-precision feature extraction of spatial information. Furthermore, Feng et al. proposed UTransNet, integrating Transformer into the U-Net architecture [25], surpassing previous U-Net-based methods on the ATLAS dataset. While Transformer-based approaches outperform traditional CNN methods, their high computational overhead and complexity, particularly in 3D medical image datasets, significantly increase training and inference time. Thus, there is a need to develop a stroke lesion segmentation method that harnesses the feature extraction capabilities of CNNs while leveraging the Transformer’s ability to capture long-range dependencies, minimizing computational overhead.

### 2.2 Medical image segmentation using multimodal fusion

Most common multimodal medical image fusion strategies use input-level fusion, where multimodal images are fused channel-by-channel as multichannel inputs to learn the fused features. Wang et al. [26] segmented brain tumors by using the four modalities in the BraTS dataset (T1, T1c, T2, and Flair) as multiple channels in the input space. Zhou et al. [27] proposed a multitasking segmentation network for channel-by-channel fusion of input multimodal MR images and brain tumors segmentation as three independent tasks. However, existing input-level fusion strategies still lack effective mechanisms to capture the semantic dependencies between different modalities.

### 2.3 Mamba for medical image analysis

Recently, Mamba [14] has demonstrated excellent performance across various modalities such as language and audio. Its outstanding capability in fast inference and capturing long-range dependencies has encouraged many researchers to apply it to medical image field. Yue et al. initially developed MedMamba for medical image classification tasks [28], (achieved 0.999 AUC). Ye et al. [29] integrated Mamba with the Perona-Malik diffusion (PMD) [30] module for echocardiogram segmentation, surpassing other algorithms in segmentation accuracy and efficiency. ProMamba [31], which incorporates hints and Mamba techniques, is quite useful in polyp segmentation tasks with strong robust generalization capabilities, even on brand new datasets. Additionally, several researchers have enhanced the architecture of U-Net by incorporating Mamba, achieving remarkable performance in lesion organ segmentation using either CT or MRI [32–35]. However, there is limited research on the application of Mamba in stroke segmentation.

## 3. Methods

**Fig 1:**
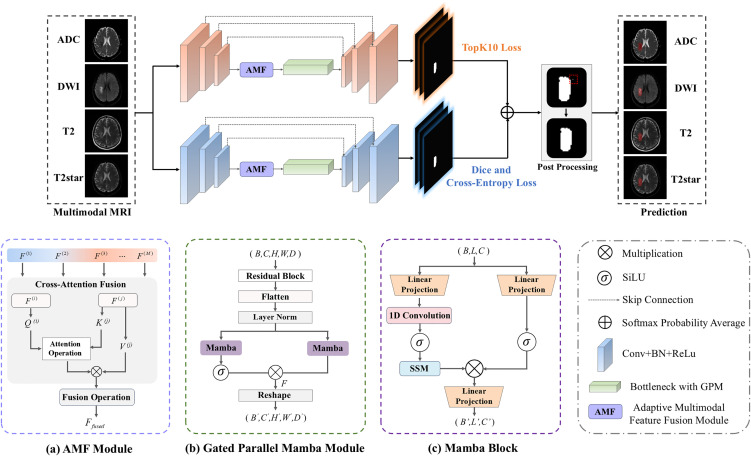
An overview of our stroke segmentation method based on the GPMNet architecture. The backbone network is based on an encoder-decoder architecture and consists of standard U-Net modules. **(a) An adaptive multimodal feature fusion (AMF) module** is introduced before the bottleneck, which dynamically integrates features from multiple MRI modalities through a cross-focusing mechanism to realize the capture of semantic information dependencies among different modalities. In bottleneck, we introduce the **(b) Gated parallel Mamba module (GPM)** to capture the long-range dependencies of the images. Each parallel branch consists of **(c) Mamba block**. The compression of feature information is achieved by gated units and parallel operations. We design and integrate two training methods to achieve generalization of the prediction results. The results after ensemble are post-processed to output the final prediction results.

In this section, we provide a comprehensive description of our stroke segmentation method, Gated Parallel Mamba Network (GPMNet), with a general overview depicted in Fig 1. The individual MRI data of the input model are used as different channel inputs to the encoder to extract features, after which the features of multiple modalities are integrated by an adaptive multimodal feature fusion module. Building the standard encoder-decoder architecture from nnUNet [36], we introduce a novel component called the gated parallel Mamba module within the bottleneck layer. This module effectively captures both local features and long-distance dependencies within the image while compressing extracted information to enhance the model’s expressiveness. To optimize performance, we devise two training strategies: one utilizing DiceBCE Loss and the other TOPK10 Loss [37]. The results obtained from these strategies are integrated and the post-processing technique of connected component analysis is applied to generate the final predictions.

### 3.1 Adaptive Multimodal Feature Fusion Module

To effectively leverage complementary information from multiple MRI modalities (e.g., DWI, ADC, T2, T2star), we propose an Adaptive Multimodal Fusion Module (Fig 1 (a)), which integrates diverse modality-specific features via a cross-attention mechanism prior to bottleneck processing. The model input as a stacked multi-modal MRI image volume:


$$\begin{array}{c} X=[X^{\left(1\right)},X^{\left(2\right)},...X^{M}]\in {\mathbb{R}}^{B\times M\times H\times W\times D} \end{array}$$ (1)

where *M* is the number of MRI modalities, *B* is batch size, *H* × *W* × *D* is image size. Each modality channel is processed independently in the encoder. Each modality is encoded using the shared nnUNet encoder to extract hierarchical feature maps, these features are collected at the deepest level (bottleneck input) for fusion.

To fuse the encoded modality-specific features $\left\{F^{\left(1\right)},F^{\left(2\right)},...,F^{\left(M\right)}\right\}$, we design an Adaptive Multimodal Feature Fusion Module (AMF) block. For two modalities *i*, *j*, the attention-weighted fusion output is:


$$\begin{array}{c} {Attn}_{i\to j}=Softmax(\frac{Q^{\left(i\right)}K^{(j)T}}{\sqrt{d}})V^{\left(j\right)} \end{array}$$ (2)

where *Q*, *K*, *V* are linearly projected features from $F^{\left(i\right)}$ and $F^{\left(j\right)}$. Final fused features are computed as:


$$\begin{array}{c} F_{fused}=\frac{\text{1}}{M}\sum_{i=1}^{M}\sum_{j=1,j\neq i}^{M}Attn_{i\to j} \end{array}$$ (3)

After that, the fused features are passed into the bottleneck Gated Parallel Mamba (GPM) module for global modeling. It is worth noting that when the input is a single modality, the AMF module changes to a constant mapping, i.e., it directly uses the deeper features of that modality as the input to bottleneck, preserving model compatibility.

### 3.2 Mamba-Based Feature Modeling and Gating Design

#### 3.2.1 Structured state space sequence models.

S4 [38] represent a recent class of sequence models employed in deep learning, closely associated with recurrent neural networks (RNNs), CNNs, and classical state space models. The S4 models are inspired by special continuous systems consisting of an implicit latent state $h\left(t\right)\in {\mathbb{R}}^{N}$ mapping a one-dimensional function or sequence x(t)∈ℝ↦y(t)∈ℝ. The S4 model comprises the parameter (Δ,A,B,C), where Δ denotes the discretization step used to transform the continuous-time system into a discrete form. The latent state $h\left(t\right)\in {\mathbb{R}}^{N}$,with *N* being the dimension of the hidden state, evolves according to:


$$\begin{array}{c} \begin{array}{c} h\text{'}\;\left(t\right)=Ah\;\left(t\right)+Bx\;\left(t\right)\\ y\;\left(t\right)=Ch\;\left(t\right) \end{array} \end{array}$$ (4)

dissociated as:


$$\begin{array}{c} \begin{array}{c} h_{t}=\overset{―}{A}h_{t-1}+\overset{―}{B}x_{t}\\ y_{t}=Ch_{t} \end{array} \end{array}$$ (5)

The parameters are transformed (Δ,A,B,C)↦(A,B,C) and the model is computed by linear recursion or global convolution:


$$\begin{array}{c} \begin{array}{c} \overset{―}{K}=(C\overset{―}{B},C\overset{―}{AB},...,C\overset{―}{A^{k}B},...)\\ y=x\overset{―}{K} \end{array} \end{array}$$ (6)

In S4, the discrete parameter (A,B,C) remains constant. Unlike S4 models, the key enhancement of the Mamba model lies in its incorporation of a selectivity mechanism. This mechanism renders the parameters in the S4 model contingent upon the inputs, enabling adaptive adjustments based on contextual content. Similar to the gating mechanism in RNNs, this feature demonstrates broader applicability within the structured state space model (SSM) framework. Through this innovation, Mamba effectively sieves out irrelevant data while preserving and amplifying task-relevant information, enhancing its capacity to capture spatial long-distance correlations. Furthermore, Mamba combines the recursive computational efficiency of RNNs with the parallel processing advantages inherent to CNNs. Leveraging the GPU’s memory hierarchy, Mamba enhances scanning operations, thereby improving computational efficiency, reducing memory overhead, and strengthening its ability to handle extensive sequences and intricate dependencies.

#### 3.2.2 Gated Parallel Mamba module.

In our model, we introduce the gated parallel Mamba module (GPM) to regulate the information flow within the Mamba module, which is depicted in Fig 1 (b). The theoretical basis for this design stems from the combination of Gated Linear Units (GLUs) [39] and the sequence modeling capabilities of State Space Models [14]. Specifically, the flattened feature vector is processed by two parallel branches, both of which employ Mamba blocks to capture global, long-range dependencies. The theoretical advantage of this dual-Mamba architecture lies in the fact that the gated mask generated by the first branch is globally context-aware. Prior to undergoing gated activation to produce dynamic weights, it processes the entire 3D sequence to comprehend the global anatomical context. When this globally informed mask is element-wise multiplied with the output sequence of the second Mamba block, the module functions as a semantic information bottleneck. In the context of stroke lesion segmentation, the role of this gating mechanism is to suppress redundant spatial information—such as healthy brain tissue and background noise—by assigning them weights close to zero. Conversely, it amplifies task-relevant semantic features—such as ischemic lesion boundaries and localized signal abnormalities—by assigning them relatively higher weights. Thus, rather than performing spatial dimensionality reduction, this module structurally condenses the feature representation.

Specifically, the image feature (B,C,H,W,D) undergoes a residual block and then a Flatten operation to obtain a vector *v* of dimension (B,H×W×D,C), denoted as (B,L,C). This vector is layer normalized and enters two branches. In one branch, the vector passes through a Mamba module and then a gating unit to derive weights ω∈(0,1),which are multiplied with the vector obtained in the other branch to produce the vector *F*, denoted as:


$$\begin{array}{c} \text{F}=\sigma \left(M\left({\text{v}}_{\text{1}}\right)\right)⊗M\left(v_{\text{2}}\right) \end{array}$$ (7)

where $v_{\text{1}},v_{\text{2}}$ refer to the same input feature vector v, which is processed in parallel by the upper and lower branches respectively. σ(·) denotes the process of generating the weights after the gating unit, and M(·) represents the computation process of the Mamba module. The vector *F* undergoes Reshape and is converted to $\left(B^{\text{'}},H^{\text{'}}\times W^{\text{'}}\times D^{\text{'}},C^{\text{'}}\right)$. Finally, it is transposed to output a vector of size $\left(B^{\text{'}},C^{\text{'}},H^{\text{'}},W^{\text{'}},D^{\text{'}}\right)$.

In addition, the architectural design of the Mamba module is depicted in Fig 1 (c). This module comprises two branches, both initiated with input feature (B,L,C), which is expanded in each branch to feature (B,2L,C). In one branch, a one-dimensional convolutional layer is followed by the SiLU activation function and a structured SSM layer. The second branch functions as a gating mechanism, applying only a SiLU activation to its input to dynamically control the flow of information. Then, one branch undergoes a multiplication operation with the other branch after the SiLU activation, projecting the feature to the size of $\left(B^{\text{'}},L^{\text{'}},C^{\text{'}}\right)$. This modular design enables seamless integration of the Mamba module into various architectures. The architecture incorporates gated connectivity and local convolutions between SSMs to enhance processing capabilities. Additionally, the architecture adopts a uniformly stacked design, enhancing the model’s flexibility and efficiency.

### 3.3 Training strategy using DiceBCE Loss

In this study, we adopt a hybrid loss strategy by combining DiceBCE Loss and TOPK10 Loss, each selected for its unique advantages in handling lesion segmentation tasks. In stroke segmentation tasks, the imbalance between foreground and background categories often leads to substantial discrepancies in segmentation outcomes. To mitigate this issue, we employ Dice Loss, which relies on Dice coefficients. Dice Loss guides the model training process by minimizing the discrepancies between Dice coefficients across different categories, thereby improving the model’s ability to differentiate between various categories during prediction. Dice Loss can be defined as follows:


$$\begin{array}{c} DiceLoss=1-\frac{2\left|X\cap Y\right|}{\left|X\right|+\left|Y\right|} \end{array}$$ (8)

where *X* denotes the set of predictions and *Y* denotes the set of Ground Truths.

In image segmentation, BCE (Binary Cross Entropy) Loss treats each pixel as an independent binary classification problem, computing the loss for each pixel based on its classification result. The BCE Loss is defined as follows:


$$\begin{array}{c} BCE=-\frac{\text{1}}{N}\sum_{i=1}^{N}\left(l_{i}\cdot log(p_{i})+(1-l_{i})\cdot log\left(1-p_{i}\right)\right) \end{array}$$ (9)

where *N* is the total number of pixels, $l_{i}$ is Ground Truth, and $p_{i}$ is the prediction result.

In our approach, one of our training strategies involves using a composite loss function comprising both Dice Loss and BCE Loss. DiceBCE Loss integrates Dice Loss and Binary Cross-Entropy Loss, which jointly optimizes both region-level overlap and voxel-wise classification accuracy. It is particularly effective in addressing class imbalance, a common issue in medical image segmentation where lesion areas are much smaller than the background. By combining these two loss functions, the model becomes more adept at learning and distinguishing between different sample categories during the training process. The hybrid loss function is defined as follows:


$$\begin{array}{c} DiceBCE=\alpha DiceLoss+(1-\alpha )BCE \end{array}$$ (10)

where α is the weight scaling factor, and α is set to 0.5 in our approach.

### 3.4 Training strategy using TOPK10 Loss

In stroke segmentation, accurately delineating small or ambiguous lesions is particularly challenging, often leading to a high number of false negatives. To address this, we incorporate a hard-mining strategy by employing TOPK10 Loss, which focuses the model’s training on the most difficult-to-classify voxels within each training sample. For each individual training sample (i.e., one 3D image-label pair in a batch), we first compute the standard cross-entropy loss for every single voxel, yielding a tensor of per-voxel loss values. TOPK10 Loss is then defined as the average of the 10 highest values from this tensor. TOPK Loss is defined as the average of the first *k* largest losses on the sample set *z*, i.e.,


$$\begin{array}{c} L_{t-k}\left(L_{z}\left(f\right)\right)=\frac{\text{1}}{k}\sum_{i=1}^{k}l_{\left[i\right]}\left(f\right) \end{array}$$ (11)

where l(f) is the cross-entropy loss and *k* is set to 10 in our approach. By backpropagating only through these “hardest” voxels, the model is encouraged to improve its performance on the most challenging regions.

While TOPK10 Loss enhances segmentation performance on challenging regions, it typically increases the training time due to the added complexity of sorting and selective backpropagation. In contrast, DiceBCE Loss facilitates faster convergence in the early training stages. Combining both allows us to balance training efficiency and fine-grained lesion detection, ultimately improving both convergence stability and segmentation accuracy.

### 3.5 Post-processing methods

After training the model using the two training strategies, inference is conducted for each sample, yielding predicted probability maps. These maps are then averaged and utilized as inputs in our post-processing method. However, in our experiments, we observed susceptibility to false positives in the segmentation results for certain samples, particularly in cases of large lesions. In such instances, small tissues like blood vessels may be inaccurately identified as lesions. To mitigate false-positive cases, we employ connected-component analysis. This method first calculates and identifies the volume of connected-component voxels *V* in the segmentation results, with the calculation formula defined as follows:


$$\begin{array}{c} V=\sum_{i=1}^{N}\sum_{j=1}^{M}\sum_{k=1}^{D}I\left(i,j,k\right) \end{array}$$ (12)

where N,M,D are the height, width and depth of the image and I(i,j,k) is the value of the binarized image voxel, which equals 1 if the pixel belongs to the concatenated domain and 0 otherwise.

**Fig 2:**
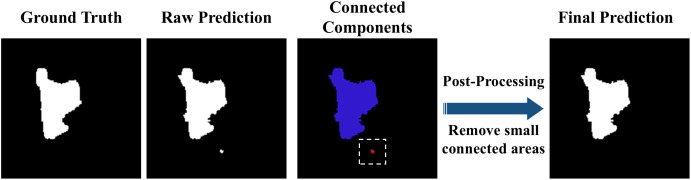
An example of our post-processing approach.

Subsequently, for each individual scan, we remove any connected component whose volume is less than 5% of the mean volume of all components identified within that specific scan—i.e., 5% of the average volume calculated by summing the volumes of all detected connected components in the scan and dividing by the total number of those components (not the largest component’s volume). This step is designed to eliminate small, isolated regions that typically represent false positives resulting from imaging noise or misclassified non-stroke tissue (e.g., blood vessels). The 5% threshold was determined empirically based on performance on our validation set. Notably, we avoided using the largest component’s volume as the reference to prevent overly strict thresholds that might accidentally exclude small but genuine ischemic lesions—addressing concerns about potential misclassification of true small lesions. We experimented with various thresholds (e.g., 3%, 5%, 8% of the mean volume) and found that 5% provided the most effective trade-off, successfully removing a majority of false positives without significantly affecting the detection of true, small ischemic lesions. This choice enhances the model’s precision while maintaining high sensitivity, thereby improving overall segmentation accuracy and clinical relevance. Fig 2 shows an example of the post-processing method we employed.

## 4. Experimental setup

### 4.1 Datasets and pre-processing

We evaluated our method using the ATLAS R2.0 [40] public dataset and our own data collected in HuanHu Hospital (Tianjin, China), labeled as HHD. The personal privacy information of patients in HHD dataset is de-identified. Therefore, this study was exempted from obtaining informed consent by the institutional research committee of the Tianjin University (Tianjin, China). The ground truth assessment of lesions in both datasets were completed by a team of board-certified radiologists and neurologists from multiple academic institutions, with inter-observer agreement verified to ensure labeling reliability. The ATLAS R2.0 dataset consists of 955 T1w MRI images, each with a size of 189 × 197 × 233 voxels, divided into 655 and 300 cases for training and test sets. The training set includes images with complete segmentation labels, while the test set consists of only images. The HHD dataset comprises 172 subjects, each containing four modalities, i.e., Apparent Diffusion Coefficient (ADC), Diffusion Weighted Imaging (DWI), T2-weighted Imaging (T2), T2-star Weighted Imaging (T2star), with the size of each image 21 × 128 × 128 voxels.

To ensure patient data consistency and mitigate noise interference, we utilized the preprocessing method in nnUNet to crop the original image data to reduce the interference of background information. Specifically, the foreground portion of the image is first identified by non-zero region threshold segmentation, and then the minimum inclusion cube is computed based on the foreground region, based on which the image is cropped to remove extraneous backgrounds and reduce the computational burden. The cropped images are further resized to a fixed size, i.e., 128 × 128 × 128 for ATLAS R2.0 images and 16 × 128 × 128 for HHD images, in order to ensure a consistent network input size.

For each case, Z-score normalization is used. This reduces the problem of inconsistent distribution between images due to differences in imaging equipment or scanning parameters, and improves the generalization ability of the model.

### 4.2 Implementation details

Our approach is based on the nnUNet framework and utilizes its self-configuration method to automatically set hyperparameters for the two datasets used in the experiments. Table 1 shows the network parameter configurations for each dataset. Our algorithms are implemented in the Pytorch framework, and all experiments are conducted on the Ubuntu 22.04.3 operating system, with environment-specific settings as shown in Table 2.

**Table 1:** Configurations for ATLAS R2.0 and HHD dataset.

Configurations	Patch size	Batch size	Epoch	Initial learning rate	Weight decay
ATLAS R2.0	(128,128,128)	2	1000	0.01	3e-5
HHD	(16,128,128)	8	800	0.01	3e-5

*ATLAS R2.0: public dataset. HHD: our own data collected in HuanHu Hospital (Tianjin, China).

**Table 2:** Experimental environment settings.

Settings	Configuration
Operating System	Ubuntu 22.04
CPU	Intel(R) Xeon(R) Silver 4210R CPU @ 2.40GHz 2.39 GHz
GPU	NVIDIA GeForce RTX 4090
Development language	Python 3.10
Development Framework	Pytorch 2.0.1

### 4.3 Evaluation metrics

To evaluate the performance of the segmentation method proposed in this paper, we adopt the evaluation metrics utilized in ATLAS R2.0 Challenge, including Dice (Dice score), VD (Volume Difference), L-F1(Lesionwise F1 Score), and SLC (Simple Lesion Count). The Dice score measures the overlap between the prediction and Ground Truth, normalizing it by the combined region’s size. It can be calculated as:


$$\begin{array}{c} Dice=\frac{2\left|P\cap T\right|}{\left|P\right|+\left|T\right|} \end{array}$$ (13)

where *P* denotes the predicted value and *T* denotes Ground Truth. Volume Difference calculates the voxel difference between the true total lesion volume and the predicted total volume, without accounting for overlap. It can be calculated as:


$$\begin{array}{c} VD=\left|V_{P}-V_{T}\right| \end{array}$$ (14)

where $V_{P}$ denotes the predicted lesion volume and $V_{T}$ denotes the true lesion volume. The Lesionwise F1 Score evaluates the detection of lesions, considering as detected if at least one voxel is predicted. It is defined as:


$$\begin{array}{c} L-F1=\frac{TP}{TP+\frac{FP+FN}{\text{2}}} \end{array}$$ (15)

where *TP* denotes true positive, *FP* denotes false positive and *FN* denotes false negative. Simple Lesion Count quantifies the disparity in the number of lesions between the predicted and Ground Truth, defined as:


$$\begin{array}{c} SLC=\left|N_{P}-N_{T}\right| \end{array}$$ (16)

where $N_{P}$ denotes the number of distinct regions in the predicted image and $N_{T}$ denotes the number of distinct regions in the Ground Truth.

## 5. Results

In this section, we present the quantitative and qualitative analyses of our method on both the ATLAS R2.0 and HHD datasets. We commence by comparing our method with several state-of-the-art methods and thoroughly analyze and discuss the review results. Subsequently, we conduct an ablation study of our method to demonstrate the effect of the AMF and GPM module on segmentation results. Additionally, we carry out ablation experiments on different training strategies and post-processing methods to showcase their impact on segmentation performance.

### 5.1 Comparison with state-of-the-art methods

**Fig 3:**
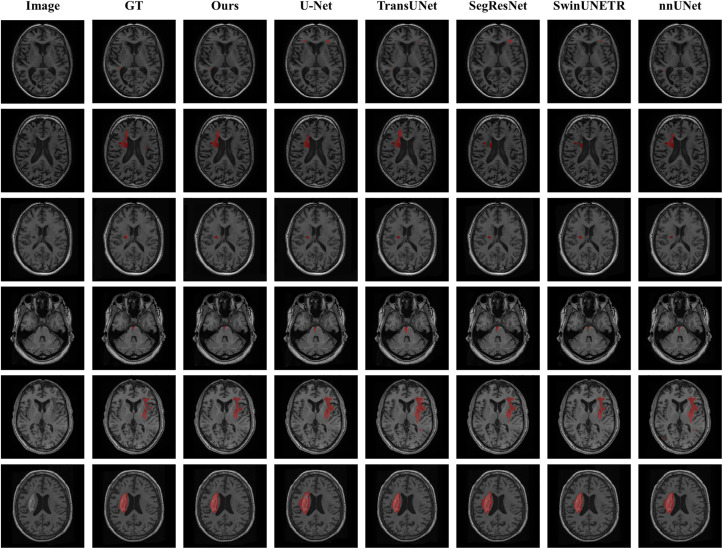
Visualization of stroke segmentation results on ATLAS R2.0 dataset. Where Image denotes the original image, GT denotes Ground Truth, and the third to sixth columns are our proposed method, UNet, TransUNet, SegResNet, SwinUNETR, nnUNet, respectively. Different lines represent different cases.

We compared five state-of-the-art methods for stroke segmentation, including U-Net [7], TransUNet [41], SegResNet [42], SwinUNETR [43], and nnUNet. To evaluate the data for both the ATLAS R2.0 and HHD datasets, we utilized a 5-fold cross-validation approach for model training and validation. Each fold of the ATLAS R2.0 dataset includes 524 cases for training and 131 cases for validation. Similarly, each fold of the HHD dataset consists of 137 cases for training and 35 cases for validation. Fig 3 shows the results of the comparison using the ATLAS R2.0 training set.

Our proposed model surpassed all the other five algorithms in segmenting both large and small lesion regions. Particularly in the segmentation of large lesions, our post-processing method effectively eliminated false-positive regions from the prediction results (e.g., line 5).

**Fig 4:**
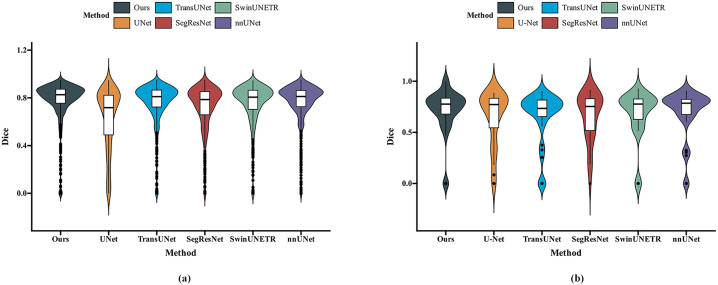
The results of the sample distribution schematic Dice score comparison between our proposed method and other state-of-the-art methods on (a) ATLAS R2.0 and (b) HHD dataset.

To further evaluate segmentation effectiveness, we compared the Dice scores obtained by our method with those of other state-of-the-art algorithms on the training set. Higher Dice coefficients indicate closer alignment between predictions and labels, indicating a greater overlap. The violin plot in Fig 4 (a) illustrates the comparison of Dice scores among different methods. Our method achieved optimal segmentation across the entire dataset compared to the other five methods. Fig 4 (b) presents the Dice values obtained by the model on the HHD dataset as a box plot. Our method again achieved the best results.

To further validate the effectiveness of our method, we submitted the prediction results of the ATLAS R2.0 test set to the official server for evaluation. A comparison analysis with the performance of other advanced algorithms is presented in Table 3. Our approach achieved a Dice score of 0.6604, a VD of 9189.04, an L-F1 of 0.5310, and an SLC of 4.6733. GPMNet achieved the best Dice score (0.6604) and the lowest SLC (4.6733), while maintaining competitive computational efficiency. Specifically, GPMNet requires 1267.53 GFLOPs and has 87.51M parameters, which is significantly lower than TransUNet (2240.26 GFLOPs, 88.62M) and nnUNet (1891.35 GFLOPs, 92.62M). Although SwinUNETR shows slightly lower FLOPs (1570.32 GFLOPs), GPMNet still achieves better segmentation accuracy with fewer resources.

**Table 3:** Results of different methods on ATLAS R2.0. The best results are shown in bold font.

Method	Dice↑	VD↓	L-F1↑	SLC↓	FLOPs (*G*)	Params (*M*)
U-Net	0.6241	10023	0.5238	5.3866	748.96	6.29
TransUNet	0.6471	9293	0.5338	4.6833	2240.26	88.62
SegResNet	0.6495	9421	**0.5351**	4.78	1158.30	18.79
SwinUNETR	0.6508	9286	0.5346	4.7666	1570.32	61.99
nnUNet	0.6540	**9184**	0.5215	4.6933	1891.35	92.62
Ours	**0.6604**	9189	0.5310	**4.6733**	1267.53	87.51

However, compared with simpler architectures like U-Net (748.96 GFLOPs, 6.29M) and SegResNet (1158.30 GFLOPs, 18.79M), our model incurs higher computational costs due to the use of stacked Mamba modules and gated parallel branches. Therefore, while GPMNet strikes a favorable balance between performance and complexity among high-performing models, its deployment in real-time or resource-constrained environments may still require lightweight model adaptation.

**Fig 5:**
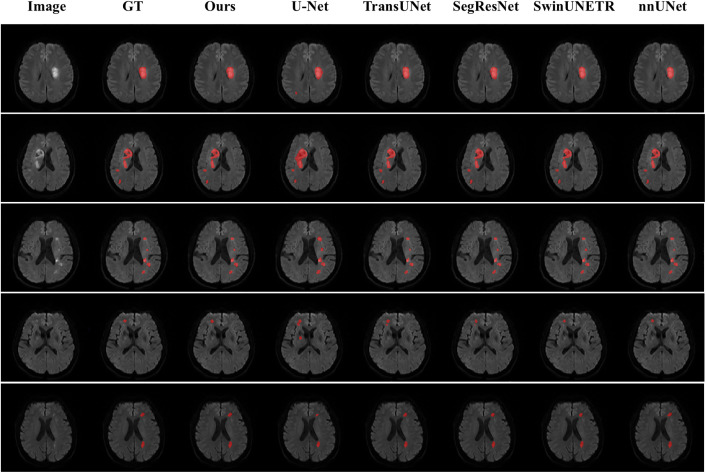
Visualization of stroke segmentation results on HHD dataset. Where Image denotes the original image, GT denotes Ground Truth, and the third to sixth columns are our proposed method, UNet, TransUNet, SegResNet, SwinUNETR, nnUNet, respectively.

**Fig 6:**
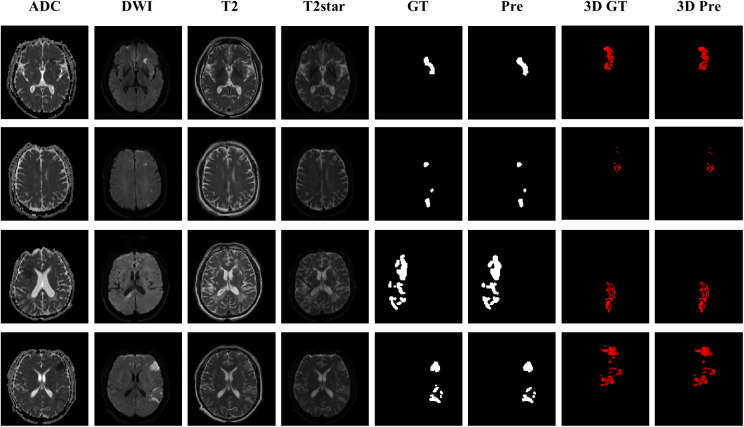
Visualization of stroke segmentation results on HHD dataset. Where ADC, DWI, T2, and T2star represent the four modalities of the magnetic resonance image, GT stands for Ground Truth, Pre represents the prediction result, and 3D GT and 3D Pre are the visualizations of their three-dimensional presentations.

In Fig 5, we compare the segmentation performance of various methods against the Ground Truth on the HHD dataset. Our approach demonstrates favorable results for diverse scenarios, including single large lesions (row 1), single small lesions (row 4), and lesions in multiple locations (rows 2, 3, and 5). Notably, for small lesions and cases with low contrast between lesion and non-lesion regions (e.g., row 4), our method accurately identifies and segments the lesion regions, whereas other algorithms may misclassify non-lesion regions as lesions, resulting in incorrect segmentation. Additionally, Fig 6 displays the 2D and 3D segmentation results of our method on selected samples from the HHD dataset. By leveraging the GPM module, our approach effectively captures the 3D features and information of each modal image, enabling precise segmentation of lesions of various shapes and sizes.

Table 4 presents the average results of our method after undergoing 5-fold cross-validation on the HHD dataset, in comparison to other state-of-the-art algorithms. Our method achieves optimal performance in the Dice, VD, and SLC metrics. Notably, it surpasses models employing Transformer structures, such as TransUNet and SwinUNETR, in several key metrics, indicating the effectiveness of our method effectively in integrating convolutional operations while capturing long-range features.

**Table 4:** Results of different methods on HHD. The best results are shown in bold font.

Method	Dice↑	VD↓	L-F1↑	SLC↓
U-Net	0.6422	5710	0.9412	1.2941
TransUNet	0.6599	5633	**0.9559**	0.8286
SegResNet	0.6583	5366	0.9321	0.8529
SwinUNETR	0.6803	5663	0.9501	1.1471
nnUNet	0.6972	5647	0.9487	1.0571
Ours	**0.7171**	**5329**	0.9527	**0.6765**

**Fig 7:**
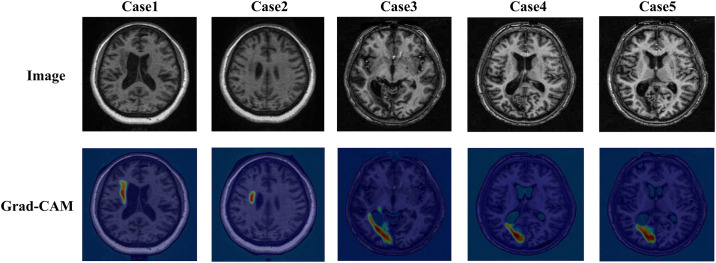
Visualization of Grad-CAM-based interpretability analysis.

Following the quantitative and qualitative comparisons, we further performed an interpretability analysis using Grad-CAM to visualize the model’s internal attention patterns. Fig 7 represents examples of Grad-CAM heatmaps derived from the final convolutional layer of GPMNet. The highlighted regions correspond to the model’s primary areas of attention during stroke lesion prediction, demonstrating that GPMNet focuses accurately on ischemic regions with high pathological relevance. Heatmap results show that GPMNet accurately focuses on clinically significant ischemic areas in various cases. This demonstrates that the proposed architecture can suppress irrelevant background responses while enhancing lesion boundaries and high-signal regions in the image. Furthermore, it exhibits biologically plausible interpretability, validating that the model’s predictions originate from physiologically consistent visual features rather than accidental associations.

### 5.2 Ablation study

We conducted ablation experiments to analyze the impact of each component of our method on the experimental results, focusing on the AMF module, GPM module, the integration of various training strategies, and the post-processing method. To evaluate the contribution of the proposed AMF module, we conducted a set of ablation experiments by selectively removing or simplifying the multi-modal fusion mechanism. The backbone network of the model is trained under the DiceBCE loss function using the nnUNet default configuration. We compared the performance of our full model on HHD Dataset with the following configurations:

- Baseline: All modalities are concatenated at the input level without semantic interaction.
- AMF w/o Attention: Modalities are averaged after encoding, removing the cross-attention structure.
- Full (AMF): Our proposed adaptive fusion module with cross-attention across modalities.

The comparison results are summarized in Table 5. The results show that early fusion strategies (Baseline) yielded suboptimal performance due to the lack of semantic interaction between modalities. When the AMF module is used without the cross-attention mechanism, only marginal improvements were observed. In contrast, incorporating the full AMF design with cross-attention across modalities significantly improves all evaluation metrics, especially Dice (0.7031) and SLC (0.8935), indicating better lesion delineation and spatial coherence.

**Table 5:** Ablation study of the AMF module on HHD dataset. The best results are shown in bold font.

Method	HHD			
	Dice	VD	L-F1	SLC
Baseline	0.6928	**5218**	0.9349	1.1200
AMF w/o Attention	0.6987	5498	0.9425	0.9562
Full (AMF)	**0.7031**	5387	**0.9463**	**0.8935**

*HHD: our own data collected in HuanHu Hospital (Tianjin, China).

This improvement can be attributed to the AMF module’s ability to adaptively model the semantic dependencies between modalities. By assigning attention-based weights to modality-specific features in a region-aware manner, the model can enhance the contribution of the most informative modality while suppressing noise or irrelevant features from others. This selective fusion leads to more accurate lesion boundaries and fewer false positives or negatives, which directly contributes to a higher Dice score. In particular, the Dice metric benefits from better overlap between predicted and ground truth masks, especially along uncertain or low-contrast lesion borders, which the AMF module helps clarify through modality consensus.

To further validate the robustness of the Adaptive Multimodal Feature Fusion (AMF) module in handling incomplete clinical data, we conducted an additional ablation study during the inference phase by systematically masking specific MRI sequences. This experiment aimed to demonstrate that the AMF module can maintain stable segmentation performance across various scenarios of missing modalities without requiring any modifications to the network architecture. The experimental results are presented in Table 6. Consistent with the configuration used in our previous AMF ablation experiments, the model was trained using the DiceBCE loss function under the default nnUNet parameter settings. To simulate missing modalities, we set the corresponding input channels to zero during inference. This was done strictly without retraining the model or altering its architecture.

**Table 6:** Performance (Dice Score) of the AMF module under various missing modality scenarios on the HHD dataset. The best results are shown in bold font.

Modalities Present	Missing Modalities	Dice
ADC, DWI, T2, T2star	None	**0.7031**
ADC, DWI, T2	T2star	0.7022
ADC, DWI, T2star	T2	0.7028
ADC, T2, T2star	DWI	0.6971
DWI, T2, T2star	ADC	0.7004
ADC, DWI	T2, T2star	0.7017
DWI only	ADC, T2, T2star	0.6992

*HHD: our own data collected in HuanHu Hospital (Tianjin, China).

As shown in Table 6, the AMF module demonstrates exceptional robustness to missing modalities without requiring any architectural modifications. Dropping auxiliary sequences (T2 or T2star) results in a negligible performance decrease, as the cross-attention mechanism seamlessly reallocates its focus to the core modalities. The most significant drop occurs when the critical DWI sequence is omitted (Dice decreases to 0.6971), directly aligning with its indispensable clinical role in acute stroke detection. Importantly, the model maintains a competitive Dice score of 0.6992 even when relying solely on the standalone DWI sequence. This tight performance fluctuation quantitatively validates that the AMF module possesses a highly stable and adaptive fault-tolerance mechanism for incomplete clinical imaging data.

To isolate and evaluate the specific contribution of the GPM module, we conducted an ablation study on both the ATLAS R2.0 and HHD datasets. The comparison results are summarized in Table 7. We compared our proposed GPMNet against a baseline model, referred to as the “Backbone,” which uses the default U-Net architecture within nnUNet. To ensure a fair and direct comparison focused solely on the architectural change, both models were trained under an identical, non-ensemble setup using only the DiceBCE loss function. All other hyperparameters, including the number of epochs (1000), were kept consistent as detailed in Table 1.

**Table 7:** Ablation study of the GPM module on ATLAS R2.0 and HHD dataset. The best results are shown in bold font.

Method	ATLAS R2.0				HHD			
	Dice	VD	L-F1	SLC	Dice	VD	L-F1	SLC
Backbone	0.6417	**9285**	0.5180	4.8233	0.6928	5218	0.9349	1.1200
GPMNet	**0.6495**	9421	**0.5351**	**4.7833**	**0.7052**	**5157**	**0.9490**	**0.9615**

*ATLAS R2.0: public dataset. HHD: our own data collected in HuanHu Hospital (Tianjin, China).

We further assess the performance of GPMNet with varying training strategies on the ATLAS R2.0 and HHD datasets. These methods were evaluated: training with DiceBCE Loss, training with TOPK10 Loss, and fusing the results obtained from these two strategies. To ensure a fair comparison, all models were trained until convergence, with the learning rate, batch size, and number of epochs set according to the automatic configurations from the nnUNet framework, as detailed in Table 1 (1000 epochs for ATLAS R2.0 and 800 epochs for HHD). The results are presented in Table 8. DiceBCE Loss addresses the class imbalance and binary classification issues, while TOPK10 Loss is more robust for small lesion segmentation. By combining the results from these two training strategies, we observed improvements in the Dice and L-F1 metrics on the ATLAS R2.0 dataset, and enhancements in the Dice, L-F1, and SLC metrics on the HHD dataset. This indicates that such an ensemble approach can partially mitigate segmentation challenges arising from variations in stroke lesion sizes and sample imbalances.

**Table 8:** Ablation study of the ensemble strategy on ATLAS R2.0 and HHD dataset. The best results are shown in bold font.

Method	ATLAS R2.0				HHD			
	Dice	VD	L-F1	SLC	Dice	VD	L-F1	SLC
DiceBCE	0.6495	9421	0.5351	4.7833	0.7052	**5157**	0.9490	0.9615
TOPK10	0.6352	**9255**	0.5117	**4.6433**	0.6949	5745	0.9474	0.8824
Ensemble	**0.6565**	9268	**0.5355**	4.7133	**0.7123**	5714	**0.9588**	**0.64**

*ATLAS R2.0: public dataset. HHD: our own data collected in HuanHu Hospital (Tianjin, China).

To validate the effectiveness of the post-processing method, we performed experiments on the ATLAS R2.0 and HHD datasets using the GPMNet baseline, which incorporates an ensemble of DiceBCE Loss and TOPK10 Loss. As summarized in Table 9, the post-processing operation led to improvements in Dice and VD indices on both datasets compared with baseline. This indicates that the method is effective in reducing false-positive segments in most samples and enhancing segmentation accuracy to a certain extent.

**Table 9:** Ablation study of the postprocess strategy on ATLAS R2.0 and HHD dataset. The best results are shown in bold font.

Method	ATLAS R2.0				HHD			
	Dice	VD	L-F1	SLC	Dice	VD	L-F1	SLC
En-GPMNet	0.6565	9268	**0.5355**	4.7133	0.7123	5714	**0.9588**	**0.6400**
En-GPMNet + PP	**0.6604**	**9189**	0.5310	**4.6733**	**0.7171**	**5329**	0.9527	0.6765

*ATLAS R2.0: public dataset. HHD: our own data collected in HuanHu Hospital (Tianjin, China).

**Fig 8:**
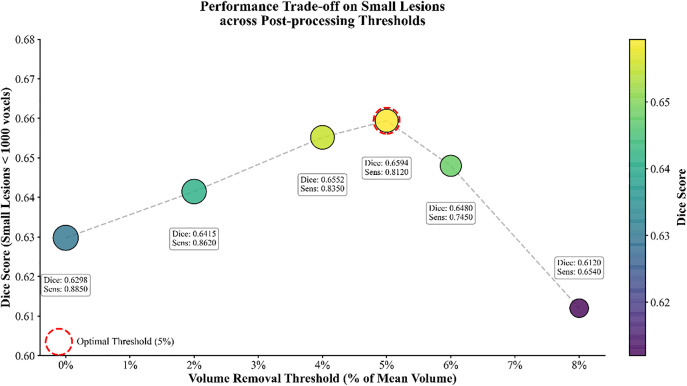
Performance trade-off on small lesions across post-processing thresholds.

To quantitatively assess the impact of post-processing thresholds on the segmentation performance for minute ischemic lesions (volume < 1000 voxels, accounting for 17% of the ATLAS R2.0 dataset) and to validate the rationale behind selecting 5% as the connected component removal threshold, we conducted a targeted ablation analysis on the ATLAS R2.0 dataset across a range of volume removal thresholds (from 0% to 8%). The performance trade-offs for the small lesion subgroup at various thresholds are illustrated in Fig 8. The results indicate that when no filtering is applied—or when the threshold is set at a low level (0%–4%)—the model maintains exceptionally high sensitivity (sensitivity > 0.8350); however, its Dice score remains constrained due to a failure to effectively filter out false-positive noise. As the threshold is raised to 6% or higher, genuine minute lesions begin to be aggressively misclassified as noise and removed, leading to a precipitous drop in sensitivity (falling to 0.6540 at the 8% threshold), which in turn severely drags down the overall Dice score (dropping to 0.6120). A threshold of 5% strikes the optimal balance, not only achieving the highest Dice score (0.6594) but also maintaining sensitivity at a high, clinically acceptable level.

## 6. Discussion

Recently, automatic segmentation of stroke foci has gained significant importance for healthcare professionals, enabling intuitive and accurate assessment of patients’ stroke conditions and treatment effects. This is essential for developing personalized treatment plans. In this study, we introduce a stroke lesion segmentation method leveraging a gated parallel Mamba structure. This structure captures the long-range dependencies in images through the Mamba module, while the gated parallel structure regulates information flow, compressing the extracted feature information and enhancing the model’s expressive power. Furthermore, we fuse the results from two training strategies to address the class imbalance and challenges in segmenting small lesions. Finally, the post-processing of prediction results aims to reduce false-positive cases. Two datasets, i.e., ATLAS R2.0 (an open-source data pool) and HHD (a private dataset collected in our hospital), were used to validate our proposed model.

Our experiments demonstrated that the proposed gated parallel Mamba structure effectively addresses the limitations of convolutional operations, capturing long-distance dependencies in images and, thereby, enhancing the accuracy of stroke lesion segmentation. Compared to other state-of-the-art algorithms, including U-Net [7], TransUNet [41], SegResNet [42], SwinUNETR [43], and nnUNet [36], our method demonstrates better performance in Dice and SLC metrics (Tables 3 and 4), achieving the highest median on the ATLAS R2.0 dataset and optimal results across the entire dataset (Fig 4). Although GPMNet achieves a favorable balance between segmentation accuracy and computational efficiency among high-performing models, it still incurs higher computational cost compared to lightweight CNN-based models such as U-Net. This may limit its direct deployment in resource-constrained clinical environments. In practice, this trade-off between accuracy and efficiency is common in medical image analysis. To further enhance practical applicability, potential strategies such as model compression, pruning, quantization, and lightweight architecture design could be employed to reduce computational overhead. These directions will be systematically investigated in our future work.

The ablation study of the AMF module (Table 5) demonstrated its effectiveness in enhancing multi-modal feature fusion. Compared with early fusion and average-based approaches, the inclusion of cross-attention in AMF yields consistent improvements in Dice, VD, L-F1, and SLC scores, highlighting its ability to capture complementary modality information. Ablation experiments (Table 7) further validate the effectiveness of our GPM module. Additionally, the visualizations of the segmentation results (Figs 3, 5 and 6) intuitively illustrate the superiority of our method. Beyond quantitative improvements, the Grad-CAM visualization (Fig 7) further confirms that the model’s focus corresponds to clinically meaningful regions, offering interpretability that facilitates potential clinical trust and adoption.

As presented in Table 8, the integration of models trained under two training strategies achieved optimal Dice and L-F1 on both datasets. Specifically, while DiceBCE Loss yielded the best results for VD on the HHD dataset and TOPK10 Loss performed best for VD and SLC metrics on the ATLAS R2.0 dataset. However, our method outperformed other approaches across different datasets and metrics, demonstrating the effectiveness of combining DiceBCE Loss and TOPK10 Loss, which leverages their individual advantages to enhance segmentation performance for lesions of varying sizes and classes.

We have also demonstrated that the post-processing step significantly enhances the accuracy of the final segmentation results. This step eliminates small-volume connected regions, thereby reducing false positives in the segmentation results. As presented in Table 9, the application of post-processing effectively improves key metrics such as Dice and VD. Notably, the VD metric indicates the disparity between the true lesion volume and the predicted volume, undergoes a decrease following post-processing. This reduction in VD metric indicates a minimization of the disparity between the predicted and true values, ultimately leading to a reduction in false positives and an enhancement in the accuracy of the segmentation results.

Although the post-processing step improves the accuracy of segmentation results by effectively removing small-volume connected regions to reduce false positives, there are some limitations of this strategy. Specifically, in the process of removing small-volume regions, some real but small-volume lesions may be mistakenly deleted, especially for the task of detecting early-stage lesions or microscopic lesions, and such mistaken deletion may lead to an increase in false-negative results, which may affect the sensitivity of the model. Thus, the post-processing strategy may reduce false positives at the expense of the recall of some small lesions. This suggests that in practical applications, the intensity of post-processing should be weighed according to the specific task requirements, or a finer-grained strategy should be introduced to distinguish real lesions from noisy regions.

There are several limitations in our study. Despite its strong segmentation performance, the clinical applicability of our method remains to be further evaluated. Future research should investigate how the model can be integrated into actual clinical workflows, such as supporting radiologists in diagnosis, assisting in individualized treatment planning, or serving as a quantitative biomarker for prognosis. Evaluating its impact on diagnostic accuracy and patient outcomes is essential for clinical translation. In addition, the modular architecture of GPMNet, especially the GPM and AMF components, suggests potential adaptability to a wide range of medical imaging tasks. This includes other imaging modalities such as CT or PET, and tasks such as tumor or organ segmentation, thereby demonstrating the model’s scalability and broader applicability. Nonetheless, several technical limitations remain. The model may have difficulty accurately segmenting extremely small lesions, which are vulnerable to being mistakenly removed during post-processing. Similarly, very large lesions with complex shapes can challenge the model’s boundary detection. Furthermore, a notable limitation of this study is that the effect of varying imaging protocols from different scanner manufacturers (e.g., differences in magnetic field strength or proprietary acquisition sequences) has not been fully examined. Although we applied Z-score normalization during preprocessing to mitigate intensity distribution shifts, the model’s performance may still degrade when applied to external datasets with distinct hardware-induced variations. Addressing this requires future validation on diverse, multi-vendor datasets and the integration of advanced domain adaptation mechanisms.

## 7. Conclusion

In this study, we proposed GPMNet, an efficient and highly accurate deep learning framework for stroke lesion segmentation. By integrating an Adaptive Multi-modal Feature Fusion (AMF) module with a Gated Parallel Mamba (GPM) structure, our approach effectively captures context-aware semantic interactions across diverse MRI modalities while efficiently modeling long-range spatial dependencies. This design successfully overcomes the localized receptive field limitations of traditional CNNs and the high computational overhead associated with Transformer architectures. Evaluated on both the public ATLAS R2.0 and our clinical HHD datasets, GPMNet demonstrated superior segmentation performance compared to state-of-the-art methods, particularly in delineating heterogeneous lesions. Furthermore, our optimized ensemble training and post-processing strategies effectively minimized false positives, while Grad-CAM analysis confirmed the clinical and physiological reliability of the model’s focus. Future work will explore incorporating additional imaging modalities and clinical metadata, alongside advancing domain-adaptive and federated learning techniques to ensure robust scalability across diverse patient populations and scanning protocols [44].
